# Effects of Psychotropic Agents on Extinction of Lever-Press Avoidance in a Rat Model of Anxiety Vulnerability

**DOI:** 10.3389/fnbeh.2014.00322

**Published:** 2014-09-15

**Authors:** Xilu Jiao, Kevin D. Beck, Amanda L. Stewart, Ian M. Smith, Catherine E. Myers, Richard J. Servatius, Kevin C. H. Pang

**Affiliations:** ^1^Neurobehavioral Research Laboratory, Veteran Affairs New Jersey Health Care System, VA Medical Center, East Orange, NJ, USA; ^2^Stress and Motivated Behavior Institute, Rutgers – New Jersey Medical School, Rutgers Biomedical and Health Sciences, The State University of New Jersey, Newark, NJ, USA; ^3^Veterans Bio-Medical Research Institute (VBRI), VA Medical Center, East Orange, NJ, USA; ^4^Department of Neurology and Neurosciences, Rutgers – New Jersey Medical School, Rutgers Biomedical and Health Sciences, The State University of New Jersey, Newark, NJ, USA

**Keywords:** avoidance perseveration, anxiolytic, behavioral inhibition, dopamine, norepinephrine, serotonin, transporter inhibitors

## Abstract

Avoidance and its perseveration represent key features of anxiety disorders. Both pharmacological and behavioral approaches (i.e., anxiolytics and extinction therapy) have been utilized to modulate avoidance behavior in patients. However, the outcome has not always been desirable. Part of the reason is attributed to the diverse neuropathology of anxiety disorders. Here, we investigated the effect of psychotropic drugs that target various monoamine systems on extinction of avoidance behavior using lever-press avoidance task. Here, we used the Wistar-Kyoto (WKY) rat, a unique rat model that exhibits facilitated avoidance and extinction resistance along with malfunction of the dopamine (DA) system. Sprague Dawley (SD) and WKY rats were trained to acquire lever-press avoidance. WKY rats acquired avoidance faster and to a higher level compared to SD rats. During pharmacological treatment, bupropion and desipramine (DES) significantly reduced avoidance response selectively in WKY rats. However, after the discontinuation of drug treatment, only those WKY rats that were previously treated with DES exhibited lower avoidance response compared to the control group. In contrast, none of the psychotropic drugs facilitated avoidance extinction in SD rats. Instead, DES impaired avoidance extinction and increased non-reinforced response in SD rats. Interestingly, paroxetine, a widely used antidepressant and anxiolytic, exhibited the weakest effect in WKY rats and no effects at all in SD rats. Thus, our data suggest that malfunctions in brain catecholamine system could be one of the underlying etiologies of anxiety-like behavior, particularly avoidance perseveration. Furthermore, pharmacological manipulation targeting DA and norepinephrine may be more effective to facilitate extinction learning in this strain. The data from the present study may shed light on new pharmacological approaches to treat patients with anxiety disorders who are not responding to serotonin re-uptake inhibitors.

## Introduction

Anxiety disorders are the most common psychiatric disorder with a lifetime prevalence of over 15% in the U.S. (Kessler et al., 2005; Somers et al., 2006). Although the etiopathology of anxiety disorders remains elusive, the core characteristic of all anxiety disorders is pathological avoidance (American Psychiatric Association, 2000). Compared to normal strategic avoidance, psychopathological avoidance is hypersensitive to stimuli, resistant to extinction, and often results in poor productivity and inefficiency that hinder daily activity (Beck et al., 2010; Berman et al., 2010). However, therapies targeting pathological avoidance are quite underdeveloped and problematic for people suffering clinical anxiety.

Current treatment for anxiety disorder includes psychological [i.e., cognitive behavioral therapy (CBT)], pharmacological approaches, and the combination of both. Extinction-resistant avoidance is one of the target symptoms in CBT, which is largely based on changing behavior through different learning approaches (Holmes and Quirk, 2010; Nic Dhonnchadha and Kantak, 2011; Schneier, 2011; Melo et al., 2012). Clinical evidence shows that combined approaches yield the highest success rate compared to each approach alone [for review, see Pollack et al. (2008)]. Among the Food and Drug Administration (FDA) approved anxiolytic agents, selective serotonin re-uptake inhibitors (SSRIs) are the drugs of first choice due to their mild side effects allowing for better compliance in patients. However, a large group of patients do not respond to SSRI treatment (>40%) or relapse after initial effective treatment (Pollack et al., 2006, 2008), providing a need for new therapeutic agents and strategies for refractory cases.

A better understanding of the neuropathology of core anxiety symptoms is essential for developing more effective treatments. We recently described a rat model of anxiety-like behavior, the Wistar-Kyoto (WKY) rat (Servatius et al., 2008; Jiao et al., 2011b). This inbred rat strain differs from normal outbred strains, such as Sprague Dawley (SD) rats, in avoidance propensity and perseveration and neuronal activity in brain regions critical for fear learning and anxiety (Beck et al., 2011; Jiao et al., 2011b). The WKY strain also exhibits behavioral inhibition temperament in the face of social and non-social stressful stimuli, heightened physiological and neuroendocrine responsiveness to stressful stimuli, and negative bias toward external cues, indicating greater anxiety vulnerability compared to normal outbred strains (Athey and Iams, 1981; Pare, 1992a,b; Redei et al., 1994; Lopez-Rubalcava and Lucki, 2000).

Although the exact mechanism for the altered behavior of the WKY rats is not well understood, much focus has been directed toward malfunction of central monoaminergic systems. The WKY rat exhibits altered dopamine (DA) and norepinephrine (NE) receptors and transporter levels in cortical and sub-cortical regions compared to SD and Wistar (WIS) rats (Jiao et al., 2011b). Pharmacological studies demonstrate that repeated treatment with drugs that enhance catecholaminergic transmission reverses abnormal behavior in WKY but not in outbred rats (Pare et al., 2001; Tejani-Butt et al., 2003). For instance, bupropion (BUP) [dopamine transporter (DAT) inhibitor] increases locomotion in the open field test (OFT), while nomifensine [DAT and norepinephrine transporter (NET) inhibitor] and desipramine (DES) (tricyclic antidepressant that mainly block NET) facilitate OFT activity and swimming behavior in the Forced Swim Test (FST) of WKY rats (Pare et al., 2001; Tejani-Butt et al., 2003). However, neither fluoxetine nor paroxetine (PAR) (selective serotonin transporter inhibitors, SSRIs) are effective on similar behaviors (Durand et al., 1999; Lopez-Rubalcava and Lucki, 2000; Tejani-Butt et al., 2003). The data suggest that anxiety-like symptoms in WKY rats are SSRI-resistant but may be modified by psychotropic drugs acting on NE and/or DA (Lahmame et al., 1997; Tejani-Butt et al., 2003).

In the present study, we compared the effects of monoaminergic transporter inhibitors on avoidance extinction in SD and WKY rats. We predicted that NET and DAT inhibitors but not SSRI would facilitate avoidance extinction and reduce active-avoidance behavior selectively in WKY rats.

## Materials and Methods

### Animals

Forty male SD (body weight = 321 ± 2.8 g) and 40 male WKY (body weight = 239 ± 1.8 g) rats (approximately 60 days of age at the start of the experiment) were obtained from Harlan Sprague-Dawley Laboratories (Indianapolis, IN, USA). Rats were housed in individual cages with free access to food and water in a room maintained on a 12:12 h day/night cycle for 2 weeks prior to experimentation. Experiments occurred between 07:00 and 15:00 h in the light portion of the cycle. One WKY rat treated with DES was eliminated from the study due to significant weight loss in the last three extinction sessions. All procedures received prior approval by the Institutional Animal Care and Use Committee at the VA New Jersey Health Care System and were conducted in accordance with the NIH Guide for the Care and Use of Laboratory Animals.

### Lever-press escape/avoidance training

The apparatus was described previously (Servatius et al., 2008). Training was conducted in 16 identical operant chambers (Coulbourn Instruments, Langhorn, PA, USA). Each operant chamber was enclosed in a sound-attenuated box. Scrambled 2.0-mA foot-shocks were delivered through the grid floor (Coulbourn Instruments, Langhorn, PA, USA). Despite an increased sensitivity to stress in WKY rats, WY, and SD rats exhibited a similar threshold to vocalize in response to foot-shock (un-published observation), suggesting similar pain sensitivity to foot-shocks. The auditory warning signal was a 1000-Hz, 75-dB tone (10 dB above background noise). A 3-min intertrial interval (ITI) was explicitly signaled with a 5-Hz blinking cue light (safety signal) located above the lever. Graphic State Notation software (v. 3.02, Coulbourn Instruments, Langhorn, PA, USA) controlled the stimuli and recorded responses.

Each session began with a 60-s stimulus-free period. A trial commenced with the presentation of the auditory warning signal. During avoidance acquisition training, a lever-press during the first 60 s of the warning signal constituted an “avoidance” response, terminated the warning signal, and triggered the ITI period. In the absence of a lever-press in the first 60 s of the warning signal, 0.5 s foot shocks were delivered with an inter-shock interval of 3 s. A lever-press during the shock period constituted an “escape response,” terminated the shock and warning signal and triggered the ITI. A maximum of 100 foot-shocks could be delivered on each trial. During avoidance extinction training, foot-shock was not delivered and safety signal was not presented. A lever-press made during the first 60 s of the warning signal constituted an avoidance response, while the lever-press made during the rest warning signal constituted an escape response. Both responses terminated the warning signal and initiated a non-signaled ITI period. Each session consisted of 20 trials. Extinction training occurred in the same training box as acquisition learning.

### Drug administration

Bupropion hydrochloride (a DAT blocker, 20 mg/ml/kg, i.p., Sigma-Aldrich, St Louis, MO, USA), desipramine hydrochloride (a NET blocker, 10 mg/ml/kg, i.p., Sigma-Aldrich, St Louis, MO, USA), paroxetine hydrochloride (a SSRI, 10 mg/ml/kg, i.p., Toronto Research Chemicals, Toronto, ON, CA), or saline vehicle solution (1 ml/kg, i.p.) was injected daily between 16:00 and 17:00 (after the avoidance session on days of training) to avoid acute drug effects on behavioral testing. The dosage used was the effective dosage tested in open field and FST on WKY rat (Tejani-Butt et al., 2003; Jiao et al., 2006).

### Sequence of behavioral procedures

Avoidance training sessions occurred three times per week (every 2–3 days). Avoidance acquisition training continued for 12 sessions. After the acquisition phase, rats were administered BUP, DES, PAR, saline (SAL) treatment, or no treatment. For each strain, rats were stratified on avoidance performance during acquisition session 12 (A12) and then randomly assigned within each stratum to BUP, DES, PAR, SAL treatment, or no injection group. Rats were treated daily from the day following the last acquisition session (A12) to the day before the sixth extinction session (E06). Extinction training (absence of shock and intertrial-interval signal) began 2 weeks after the last acquisition session (session 13) and continued for nine sessions. Therefore, the first six sessions of extinction were with drug or SAL and the last three extinction sessions were drug-free. Thus, extended drug effect was evaluated in the last three drug-free sessions.

### Data analysis

Mixed design analysis of variance (ANOVA) was used to analyze behavioral aspects of acquisition and extinction. Ratios of avoidance and escape responses, lever-presses during the first minute of each session [anticipated responses (AR)] and non-reinforced intertrial-interval responses (ITRs) during the first, second, and third minute ITI (ITI-1, ITI-2, and ITI-3 min) were examined in both phases. Both between-session and within-session avoidance responses were examined to illustrate the main effects of strain, drug treatment, and interactions.

In the acquisition phase, two-way ANOVA with repeated measures of session and between-groups measure of strain (2 × 12) was conducted to analyze all behavioral features using Tukey-Kramer for *post hoc* comparisons. Within-session avoidance responses were examined in early (A01–04), mid (A05–08), and late (A09–12) session-blocks with four sessions/block across trials (2 × 20).

In the extinction phase, mixed design ANOVA was used to analyze all the behavioral aspects. Analysis of rats receiving SAL injection compared to non-injection animals revealed no differences (all *F*-values <1 and *p*-values >0.2), and so data from subjects in these two groups were combined into one control (CTL) group within each strain for analysis and figure illustration during extinction. A mixed ANOVA with between-subjects factors of strain and treatment and repeated measures of session (2 × 4 × 9) was used to analyze main factors of strain, treatment, session, and interactions. In order to examine the immediate (i.e., when treatment was administered) and lasting (i.e., when treatment was discontinued) drug effects on extinction learning in each strain, the mean avoidance responses were separately analyzed within the first six sessions and the last three sessions of extinction, using two-way ANOVA with repeated measures of session and between-groups measure of treatment design, treatment × extinction session (4 × 6 and 4 × 3, respectively). To evaluate drug effects on within-session extinction learning, within-session avoidance response was analyzed in early (E01–03), mid (E04–06), and late (E07–09) extinction, respectively, using strain × treatment × trial (2 × 4 × 20) design with strain and treatment as between-subjects factors and trial as a within subject factor. *Post hoc* analysis was conducted using Dunnett’s test to identify interactions.

All data are expressed as means ± SEM. An alpha level equal to 0.05 was used to determine significance across all analyses. Statistical results are reported only where significant differences were found.

## Results

### Acquisition

#### Avoidance responding

**Figure 1:**
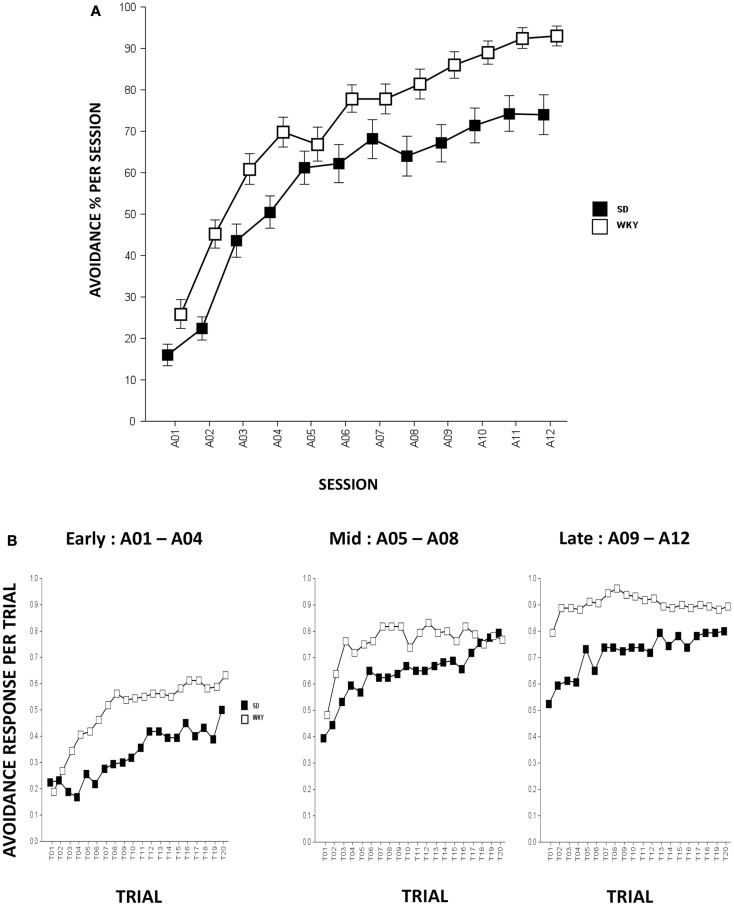
**Avoidance response during acquisition**. **(A)** Avoidance lever-press responding significantly increased in both strains although WKY rats acquired avoidance responses significantly faster and reached greater asymptotic performance compared to SD rats. **(B)** Within-session avoidance response. Both strains emitted more avoidance responses as an acquisition session proceeded. During early, mid, and late acquisition phases, WKY rats exhibited significantly faster within-session avoidance acquisition compared to SD rats. SD rats emitted less avoidance responding in the first trial of a session compared to the last trial of a previous session; however, this phenomenon is not evident in WKY rats. Each data point represents group mean ± SEM (*n* = 40/strain).

In all respects, strain differences in avoidance learning in this study replicate what has been described previously (Servatius et al., 2008; Beck et al., 2010, 2011). Rats from both strains emitted greater numbers of avoidance responses as acquisition proceeded, Session, *F*(11,858) = 101.8, *p* < 0.001 (Figure 1A). Compared to SD rats, WKY rats acquired avoidance response to a greater extent, strain, *F*(1,78) = 17.8, *p* < 0.001.

Within-session analysis was conducted to compare avoidance responses in three-session blocks (i.e., early, mid, and late blocks). Within-session avoidance responses are averaged across early (A01–04), mid (A05–08), and late (A09–12) acquisition sessions. The data indicate that both strains emitted more avoidance responses in later trials of the session, Trial, *F*(19,1482) = 23.3 (early, sessions A01–04), 13.9 (mid, sessions A05–08), and 7.2 (late, sessions A09–12), *p*s < 0.001. WKY rats exhibited superior within-session avoidance learning compared to SD rats, strain, *F*(1,78) = 24.8 (early), 5.6 (mid), and 15.2 (late), *p*s < 0.001. Consistent with our previous findings, the within-session acquisition learning is more obvious in SD rats as WKY rats emitted similar or greater avoidance responding on the first trial of a session compared to the last trial from the previous session, suggesting a lack of “warm-up” that plays a pivotal role in the development of avoidance perseveration during extinction phase in the WKY strain (Servatius et al., 2008) (Figure 1B).

#### Non-reinforced response

In terms of ARs, WKY rats made more lever-presses during the first minute of each session as compared to SD rats, strain, *F*(1,78) = 4.3, *p* < 0.05; both strains of rats emitted more responses as acquisition proceeded, session, *F*(11,858) = 18.2, *p* < 0.001 (Figure 4A). The number of intertrial-interval responses (ITRs) in the first, second, and third-minute of the ITI period was altered as the acquisition phase proceeded, *F*(11,858) = 24.4 (ITI-first minute), 13.0 (ITI-second minute), and 14.5 (ITI-third minute), *p*s < 0.001 (Figure 3A). Both strains of rats emitted more ITRs in the first minute compared to the second and third minute. ITRs differed between strains for all ITIs, strain × session, *F*(11,858) = 16.3 (ITI-first minute), 11.60 (ITI-second minute), and 15.1 (ITI-third minute), respectively, *p*s < 0.001; WKY rats responding more frequently in early than late acquisition sessions, whereas SD rats emitted similar number of ITRs across acquisition sessions.

### Extinction

#### Avoidance responding

**Figure 2:**
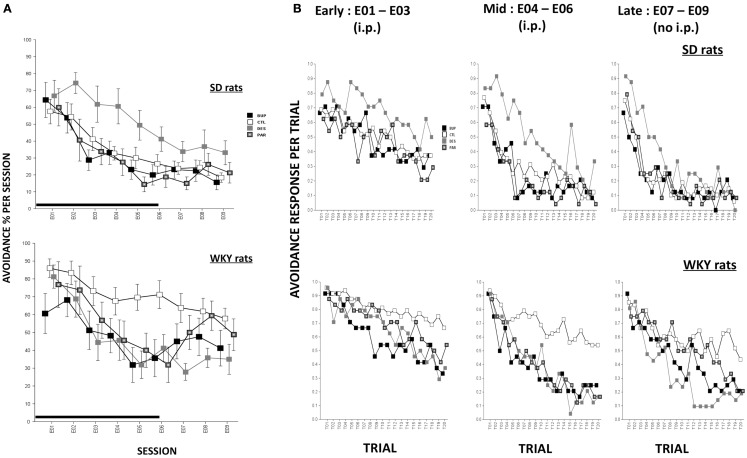
**Avoidance response during extinction**. **(A)** Avoidance response decreased in both strains while WKY rats extinguished slower as compared to SD rats in general. WKY CTL rats emitted significantly more avoidance responses than SD CTL rats. In WKY rats, BUP treatment significantly decreased avoidance responses during injection sessions while DES treatment significantly reduced such responses during all extinction sessions, compared to CTL group. In SD rats, DES treatment impaired extinction of avoidance response compared to CTL. **(B)** Within-session avoidance response. WKY rats emitted significantly more avoidance responses than SD rats. In WKY rats, all three drugs facilitated within-session extinction in the mid sessions compared to CTL treatment. DES treatment maintained extinction facilitation in late sessions while no injection was administered. In contrast, in SD rats, DES treatment trended to produce increased avoidance response in mid and late extinction sessions compared to CTL. Each data point represents group mean ± SEM. (*n* = 8/treatment group, 16/CTL group). Gray shade on *x*-axis indicates sessions in which drugs were administered (E01–06).

During extinction, all rats made fewer avoidance responses as extinction proceeded across sessions, *F*(8,573) = 47.96, *p* < 0.001 (Figure 2A). Overall, WKY rats emitted more avoidance responses compared to SD rats, *F*(1,72) = 14.88, *p* < 0.001. Similar to our previous findings, WKY rats without drug treatment exhibited more avoidance responses compared to SD rats without drug, as reflected by significant strain × treatment interaction, *F*(3,72) = 3.91, *p* < 0.05. In an analysis of only WKY rats, DES treatment facilitated extinction compared to the un-drugged (CTL) group as reflected by treatment × session, *F*(24,285) = 2.01, *p* < 0.005, *post hoc p* < 0.05; in contrast, DES treatment in SD rats enhanced avoidance responses compared to CTL as reflected by treatment, *F*(3,36) = 3.48, *p* < 0.05, *post hoc p* < 0.05.

Rats were treated with drug or SAL for the first six sessions of extinction, and then untreated for another three sessions of extinction. In order to assess immediate drug effects and extended drug effects on extinction learning in both strains, mean avoidance responses were compared between treatment groups within each strain for extinction sessions with treatment (E01–06) and extinction sessions without treatment (E07–09).

#### Extinction sessions with treatment (E01–06)

During the first six extinction sessions, all rats reduced avoidance response as extinction proceeded, reflected by main effect of session, *F*(5,360) = 52.76, *p* < 0.001; WKY rats emitted more avoidance responses compared to SD rats, as reflected by a main effect of strain, *F*(1,72) = 8.96, *p* < 0.005, and a strain × treatment interaction, *F*(2,72) = 3.36, *p* < 0.05. In a separate analysis of WKY rats, BUP treatment decreased avoidance responding in all extinction sessions except E02, DES treatment decreased avoidance responding in sessions E03–06, and PAR treatment decreased avoidance responding in the last two extinction sessions, as reflected by treatment × session interaction, *F*(15,180) = 2.31, *p* < 0.01, *post hoc*, *p*s < 0.05, suggesting all three drugs facilitated extinction when daily treatment was administered. In an analysis of SD rats, DES treatment led to the highest number of avoidance response among all treatment groups; the remaining groups (i.e., BUP, PAR, and CTL) did not differ, main effect of treatment, *F*(3,36) = 3.41, *p* < 0.05, *post hoc p* < 0.05. These results suggest DES treatment is detrimental for extinction learning in SD rats.

#### Extinction sessions without treatment (E07–09)

During the last three sessions of extinction when treatment was discontinued, WKY CTL rats emitted more avoidance response compared to SD CTL rats [strain × treatment, *F*(3,71) = 4.0, *p* < 0.05]. In the analysis of WKY rats, DES group exhibited a trend of less avoidance responses compared to CTL group as reflected by main effect of treatment that just missed significance (*p* = 0.066). In the analysis of SD rats, DES group did not differ from other treatment groups when the drug administration was discontinued; suggesting that enhanced avoidance following DES treatment (E01–06) was not long-lasting.

Within-session avoidance responses are as averaged across early (E01–03), mid (E04–06), or late (E07–09) extinction sessions. Within-session analysis demonstrated that avoidance responses decreased significantly in both strains with increasing trials in a session, *F*(19,1368) = 23.07 (early, sessions E01–03), 46.45 (mid, sessions E04–06), and 44.75 (late, sessions E07–09), *p*s < 0.001 (Figure 2B). CTL WKY rats exhibited significantly higher avoidance responses compared to CTL SD rats throughout mid and late sessions indicating slower within-session extinction in WKY rats without drug treatment, *F*(3,72) = 4.89 (mid) and 4.00 (late), *p*s < 0.005 and 0.05, respectively, *post hoc p*s < 0.05. However, in both strains, drug treatment affected avoidance responding differently in early, mid and later session-blocks. In WKY rats, BUP and DES significantly facilitated within-session extinction during early, middle, and late phases of extinction compared to CTL (early: treatment × trial interaction, *F*(57,684) = 1.47, *p* < 0.05; middle: *F*(3,36) = 4.31, *p* < 0.05; late: *F*(57,684) = 1.75, *p* < 0.001. More over, first trial avoidance was not altered regardless of treatment, suggesting between-session extinction may not be apparent measured by first trial avoidance. In SD rats, none of the drugs affected avoidance response in early extinction sessions. However, in mid and late phases of extinction, DES impaired within-session extinction (enhanced avoidance responding) compared to the other treatments [middle: main effect of treatment, *F*(3,36) = 5.0, *p* < 0.005, and treatment × trial interaction, *F*(57,684) = 1.51, *p* < 0.05, *post hoc p*s < 0.05; late: treatment × trial interaction, *F*(57,684) = 1.45, *p* < 0.05, *post hoc p*s < 0.05]. Neither BUP nor PAR significantly altered within-session extinction in SD rats.

#### Non-reinforced responding

**Figure 3:**
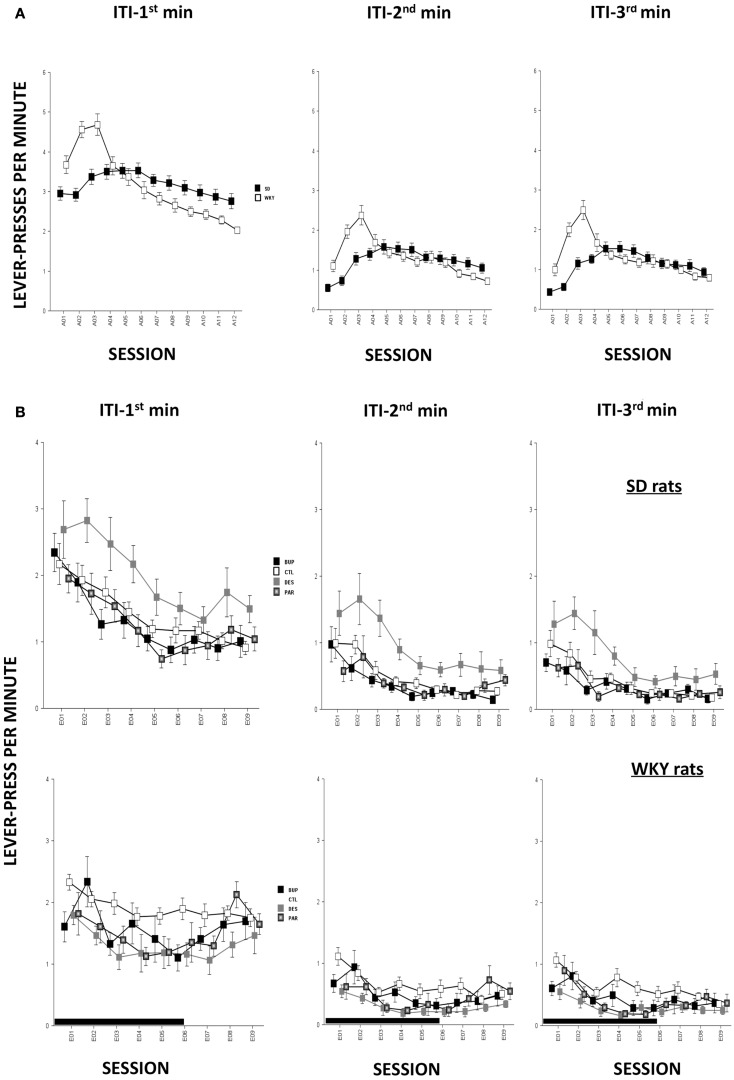
**Non-reinforced responses (ITRs)**. ITRs within each 1-min window are depicted in separate panels. More ITRs were performed during the ITI-first minute compared to the ITRs performed during the ITI-second and ITI-third minute regardless of strain or treatment. **(A)** During early acquisition, WKY rats emitted more ITRs than SD rats, and more ITRs compared to late acquisition sessions. However, SD rats emitted relatively constant numbers of ITRs during the ITI-first minute as acquisition proceeded and more ITRs during ITI-second and -third minute windows in mid acquisition sessions. **(B)** During extinction, DES facilitated ITRs selectively in SD rats across all three ITI windows compared to CTL. None of the treatments affected ITRs in WKY rats regardless of ITI windows. Each data point represents group mean ± SEM. (*n* = 8/treatment group, 16/CTL group). Gray shade on *x*-axis indicates sessions in which drugs were administered (E01–06).

**Figure 4:**
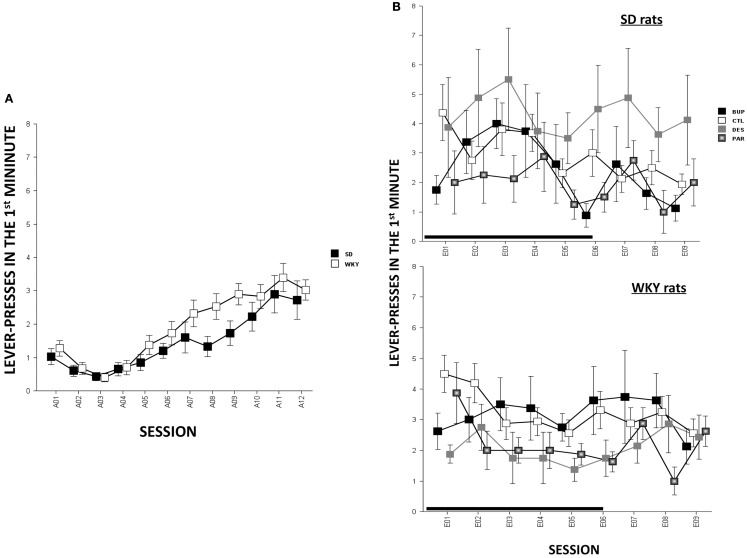
**Lever-press during the first minute of each session [i.e., anticipated response (AR)] in SD and WKY rats**. **(A)** During the acquisition phase, WKY rats emitted more ARs than SD rats. Both strains increased ARs as acquisition continued. **(B)** During the extinction phase, the ARs did not change as extinction continued regardless of strain or treatment. However, during late extinction sessions (E07–09), DES-treated SD rats emitted more ARs than the other SD groups. Each data point represents group mean ± SEM (*n* = 8/treatment group, 16/CTL group). Gray shade on *x*-axis indicates sessions in which drugs were administered (E01–06).

Anticipated responses decreased during the extinction phase, *F*(8,573) = 2.09, *p* < 0.05 (Figure 4B). Notwithstanding that ARs in WKY rats were not affected by any drug treatment, DES treatment increased lever-presses compared to CTL treatment in SD rats, *F*(3,36) = 3.11, *p* < 0.05, *post hoc p* < 0.05. On the other hand, fewer lever-presses were emitted by all rats in each minute of the ITI as extinction proceeded as reflected by main factor of Session, *F*(8,573) = 31.5 (ITI-first minute), 26.25 (ITI-second minute), and 29.78 (ITI-third minute), respectively, *p*s < 0.001 (Figure 3B). More ITRs were emitted during the ITI-first minute compared to responses emitted during the second and third minute regardless of strain or treatment. In WKY rats, DES-treated rats emitted fewer ITRs than CTL-treated peers, *F*(24,285) = 2.47 (ITI-first minute), 1.89 (ITI-second minute), and 1.76 (ITI-third minute), *p*s < 0.05, *post hoc*, *p*s < 0.05. In contrast, DES treatment in SD rats enhanced ITRs compared to the other treatments, *F*(3,36) = 3.07 (ITI-first minute), 6.40 (ITI-second minute), and 5.14 (ITI-third minute), *p*s < 0.05, *post hoc*, *p*s < 0.05.

## Discussion

Avoidance and its perseveration represent key features of anxiety disorders. Pharmacological approaches that reduce avoidance behavior could facilitate recovery in patients with anxiety disorders. The present study used an animal model of behavioral inhibition, a risk factor for anxiety disorders, to test the effectiveness of pharmacological intervention on preservative avoidance behavior. WKY rats exhibited facilitated acquisition and delayed extinction of lever-press avoidance compared to SD rats, consistent with our previous reports (Servatius et al., 2008; Beck et al., 2010, 2011; Jiao et al., 2011a). Moreover, drugs targeting distinct monoamine systems affected extinction learning differently and in a strain-dependent manner. BUP and DES significantly facilitated extinction of avoidance selectively in WKY rats while none of the drugs enhanced extinction learning in SD rats. Instead, DES impaired extinction learning in SD rats by increasing avoidance responding. These data suggest that drugs can facilitate extinction of avoidance response selectively in animals with innate vulnerability to anxiety.

In addition, the sensitivity to pharmacological manipulations was not necessarily similar between different behavioral measures. For instance, BUP and DES reduced avoidance responses in WKY rats without affecting ARs or ITRs. In SD rats, DES not only increased avoidance response but also enhanced ITRs. Despite the fact that AR, ITR, and avoidance responding during extinction all constitute non-reinforced behaviors, the results suggest that these behaviors may be under different neurochemical CTL and that pharmacological treatment could be designed to selectively alleviate psychopathological avoidance and leave other behavioral features intact to reduce side effects.

The results of the present study provide important information regarding the neurobiological mechanisms of extinction of avoidance behavior. Several neurochemical pathways have been implicated in the development of anxiety and its neuropsychopharmacology, while the neural mechanism of avoidance and its extinction is far less understood. Converging literatures demonstrate that an aberrant DA circuitry and (or) defective noradrenergic function is associated with anxiety disorders (Mathew et al., 1981; Hamner and Diamond, 1996; Ballenger, 2001). Early studies on the neurobiology of active avoidance also focused on catecholamine systems (Beer and Lenard, 1975; Ashford and Jones, 1976; Fibiger and Mason, 1978; Oei and King, 1978; Raskin et al., 1983; Koob et al., 1984). In general, DA has mostly been implicated in the acquisition and expression of avoidance responses (Lenard and Beer, 1975; Fibiger and Mason, 1978) while NE may be more involved in the extinction of such responses (Lenard and Beer, 1975; Fibiger and Mason, 1978; Raskin et al., 1983). However, selective lesions that targeted either the DA or NE system have yielded inconsistent results. For instance, NE depletion did not appreciably alter avoidance learning, but rather led to impairment of extinction (Lenard and Beer, 1975; Fibiger and Mason, 1978), while mice that lacked NE extinguished more rapidly compared to intact CTLs (Thomas and Palmiter, 1997). The inconsistent effects of lesions may be due to different lesion procedures and compensatory mechanisms after lesion.

Extinction deficiency has been associated with malfunction of various brain regions, especially the medial prefrontal cortex (mPFC) and amygdala. The evidence is mainly obtained from fear extinction paradigms. Hypoactive mPFC and hyperactive limbic system, including nucleus accumbens (NAc) and amygdala, are susceptibility factors in psychopathology of anxiety disorders (Milad et al., 2006; Rauch et al., 2006). Historically, dysfunctional catecholamine transmission in the mPFC and NAc has been associated with abnormal active-avoidance behavior and implicated in anxiety pathology (Giorgi et al., 1994; Duncan et al., 1996; Lacroix et al., 1998; Weiss et al., 2001). However, results in rodents are inconsistent due to the wide variety of animal models, behavioral procedures and techniques employed. Here we utilized (1) pharmacological agents that modulate NE, DA, and 5-HT neurotransmission in the brain by blocking corresponding transporters in order to identify their role in extinction of avoidance and (2) a unique rat model, the WKY rat strain that exhibits innate abnormalities in DA and NE systems.

In the present study, DES, a tricyclic antidepressant that increases synaptic NE level, facilitated extinction in WKY rats after 2 weeks of treatment. In previous studies, increased locomotion in OFT and swimming time in FST were reported following DES treatment in WKY rats (Lopez-Rubalcava and Lucki, 2000; Tejani-Butt et al., 2003). Thus, the present results with those obtained previously suggest that blocking NET can ameliorate anxiety- and depression-like behaviors in the WKY rat. WKY rats exhibit higher NE transporter (NET) binding in hippocampus and amygdala compared to SD rats, and repeated exposure to novel stressors reduced β- and α2-adrenergic receptors selectively in WKY rats, suggesting a pre-existing vulnerability to stress is associated with malfunctions in noradrenergic system (Tejani-Butt et al., 1994). In the present study, the 2 mA foot-shock during acquisition may function as repeated physical stressors, while the context and the warning signals may function as repeated psychological stressors; both could alter NET and receptor function in WKY rats, which could lead to exaggerated avoidance response. DES exerts its pharmacological effects via inhibition on NET and auto-receptor desensitization in rats (Sacchetti et al., 2001; Zhao et al., 2008; Zhang et al., 2009). Chronic DES treatment (i.e., more than 10 days) not only reduces NET binding, but also alters β- and α-adrenergic receptor binding in a region-specific manner in rats (Hancock and Marsh, 1985; Zhao et al., 2008; Zhang et al., 2009). Treatment with DES at the same dose that altered NE receptor and NET (i.e., 10 mg/kg/day) blocked stress – and alcohol-induced anxiety-like behavior in WKY rats (Durand et al., 2000; Getachew et al., 2008). Thus, DES administration was expected to improve extinction in WKY rats, possibly through pharmacological changes in the NE system. In contrast, the same treatment appeared to retard avoidance extinction in SD rats. The differential effects of DES on avoidance response in the two strains could be attributed to different innate noradrenergic function. DES-treated SD rats also emitted more non-reinforced response (i.e., ITRs) during extinction, suggesting the retardation of avoidance extinction may be due to elevated general locomotor activity, which has also been reported previously following DES treatment (Maj et al., 1987; Tejani-Butt et al., 2003). Thus, altering NE function could yield different outcomes depending upon baseline NE activity and individual variability in noradrenergic system following chronic drug administration.

Here, we also report that BUP, a selective DAT blocker and a weaker NET blocker with antagonism of adrenergic receptors and acetyl cholinergic receptors (Carroll et al., 2014), facilitates extinction of active-avoidance selectively in the WKY strain. These effects of BUP may be related to the fact that WKY rats have an altered DA system associated with the distribution of transporter and receptors in the brain (Jiao et al., 2003, 2006; Yaroslavsky et al., 2006; Novick et al., 2008; Yaroslavsky and Tejani-Butt, 2010). Given the role of the mesolimbic DA system in cognitive, emotional, and motivational behaviors, we previously examined the distribution of DAT sites in the brains of WKY compared to Wistar (WIS) and SD rats and reported that WKY rats exhibited a differential pattern of distribution of DAT binding sites in terminal field regions versus the cell body areas in comparison to WIS and SD rats (Jiao et al., 2003). At the time, we speculated that the observed differences in the density and distribution of DAT sites in WKY rats may lead to altered modulation of synaptic DA levels in the cell body and mesolimbic regions and contribute to behavioral differences previously observed. In terms of the role of DA in active avoidance, the results are not clear and may depend on which region investigated. NAc DA depletion leads to a substantial reduction in learning to lever-press to avoid or escape a shock (McCullough et al., 1993), while mPFC 6-OH-DA lesions, which reduce DA level to 13% of the CTL levels, do not affect avoidance responding (Koob et al., 1978; Sokolowski et al., 1994). Although the involvement of DA in the extinction of active avoidance is unknown, DA in mPFC and amygdala is actively involved in extinction of conditioned fear in rodents through modulation of GABAergic neurons in the intercalated cell cluster (ITC) of amygdala and basolateral amygdala (Morrow et al., 1999; Fernandez Espejo, 2003; de la Mora et al., 2010; Rey et al., 2014). WKY rats exhibit slower extinction of lever-press avoidance and lower mPFC activity and amygdalar GABAergic activity compared to SD rats (Jiao et al., 2011a), suggesting dysfunctional DA transmission in mPFC and amygdala could be the possible mechanisms. Repeated treatment with DAT blockers (i.e., BUP and nomifensine) not only increases DAT levels in mesolimbic regions (Jiao et al., 2006) but also facilitates extinction learning (in the present report) and reduces anxiety-like behavior in the OFT (Tejani-Butt et al., 2003), further supporting that DA is playing a critical role in modulating emotion and responses associated with aversive stimuli.

Nucleus accumbens, a heavily DA-innervated limbic area, is another region of interest involved in BUP-associated effects because of its role in motivated behavior and emotion (Koob, 1992; Ahn and Phillips, 2007). Higher DA turnover rate and receptor binding combined with lower DAT binding in the NAc leads to elevated DA activity in the NAc in WKY rats (Jiao et al., 2003; De La Garza and Mahoney, 2004; Novick et al., 2008; Scholl et al., 2010), and this condition is often associated with increased emotionality and greater avoidance responding (Ikemoto and Panksepp, 1999). In the present study, BUP administration accelerated extinction in WKY rats, supporting a positive DA involvement in extinction learning. Moreover, PFC DA is important for cognitive processes such as decision-making and avoidance, but PFC has very low DAT distribution in rats and the reuptake of DA in this region mainly relies on NET (Wayment et al., 2001; Moron et al., 2002). Therefore, both DES and BUP may elicit similar effects (i.e., increased synaptic DA and NE levels) within PFC, which is a possible mechanism underlying their similar effects on extinction in WKY rats.

The role of 5-HT in avoidance is less clear. Earlier pharmacological studies using one-way avoidance in shuttle box demonstrated that increased 5-HT transmission is associated with a deficit in acquisition and retention and decreased 5-HT leads to facilitated acquisition through the hippocampus and prefrontal cortex (Ogren, 1986a,b). However, pharmacological agents that facilitate serotonin transmission either impaired passive avoidance and facilitated its extinction (Shugalev et al., 2008) or had no effect on a two-way avoidance task (Sun et al., 2010) in rats. Moreover, chronic fluoxetine treatment reverses generalized avoidance in a mouse model of post-traumatic stress disorder (PTSD) (Pamplona et al., 2011), suggesting serotonergic agents modulate avoidance and its extinction via influencing the emotional response. Thus, serotonergic agents seem to be in a good position to alter behavioral abnormalities such as persistent avoidance. Most importantly, although SSRIs are the first line medication to treat anxiety symptoms, they are found to be ineffective in many patients (Pollack et al., 2006, 2008). Similar to those SSRI refractory cases, WKY rats do not respond to chronic SSRI treatment measured by OFT or FST at baseline condition or following stress challenge (Sanchez and Meier, 1997; Durand et al., 1999; Lopez-Rubalcava and Lucki, 2000; Tejani-Butt et al., 2003; Rosenzweig-Lipson et al., 2007). Here, we observed that PAR facilitated within-session extinction learning in WKY rats only in the mid extinction sessions; however, both BUP and DES facilitated within-session extinction in early, mid, and late extinction. Moreover, WKY rats treated with PAR appeared to resume avoidance responses in extinction sessions in which the drug was not on board. Therefore, the WKY rat may be a useful model for SSRI-resistant anxiety.

We also found that PAR, at a dose that is effective in reducing stress-induced abnormalities in OFT/FST in SD rats (Tejani-Butt et al., 2003), did not change avoidance responding in SD rats in the present study. This discrepancy could be due to different behavioral procedures and paradigms used in previous studies and the present study. Previously, relatively short behavioral tests such as OFT and FST were used to evaluate emotional response following various durations of stress period (i.e., acute versus 7 days to weeks of chronic stress). Here, we trained rats to acquire lever-press avoidance using foot-shock for 12 sessions and each session lasted for over an hour depending on performance. This paradigm allows the development of effective coping mechanisms in normal rat strains but promotes avoidance perseveration in rats that are vulnerable to stress, such as the WKY strain (Jiao et al., 2011a). Therefore, the lack of effect in SD rats following PAR treatment here may be explained by normal coping behaviors being more resilient to pharmacological intervention due to homeostasis in brain neurochemistry. However, the possibility that a higher dose may have facilitated avoidance extinction can not be ruled out since only a single dose was tested in the present study. Thus, our findings of the distinctive role of monoamine in avoidance behavior will, hopefully, shed a light on the neurochemical mechanisms underlying anxiety disorders.

Anticipated responses are often associated with fear and anxiety disorders in humans and experimental animals (Conrod, 2006; Bailey and Crawley, 2009; Straube et al., 2009). However, whether and how this behavioral feature responds to pharmacological manipulation has not been thoroughly studied. Consistent to our previous report (Perrotti et al., 2013), here we found that WKY rats exhibited more ARs during acquisition compared to SD rats, suggesting a positive relationship between ARs and avoidance-prone behavior. However, this strain difference disappeared during extinction in CTL-treated WKY and SD rats, suggesting ARs may be labile depending on environmental factors (i.e., both foot-shock and the flashing light were removed during extinction). In addition, none of the agents significantly altered ARs in drugged groups compared to CTL groups, regardless of strain. The present data provide little support to associate ARs with avoidance perseveration. However, evaluating ARs in anxiety is beyond the scope of this study since we only measured lever-press as the main response to evaluate AR. Physiological and autonomic responses such as skin conductance and heart beat may be more appropriate to study anticipatory responding. In the future, these measurements may be used to better characterize pharmacological effects on ARs.

Lastly, we believe that the effects of the agents used in the present study are due to chronic pharmacodynamics changes at transporter and receptor levels instead of neurochemical concentration changes at synaptic level. Given that all three agents have relatively short half-lives in rat brain tissue, from 5 h (PAR) to 8 h (DES) (Suckow et al., 1986; Caccia et al., 1993; Cox et al., 2011), we treated animals over 12 h before the start of the first post-treatment extinction session. Moreover, other post-treatment extinction sessions occurred days and weeks after the last administration, which provides sufficient clearance to elucidate non-drug effect on extinction. Further examination of neuronal activation in limbic regions will provide direct evidence illustrating how these agents affect avoidance behavior in both strains. On the other hand, only one dosage for each agent was used in this study. Although SSRIs have a relative flat dose–response curve to treat social anxiety disorder and fixed-dose of SSRIs has been used as a standard treatment strategy, clinical evidence suggests that optimal effect may be obtained with higher doses of SSRI (van der Linden et al., 2000; Baker et al., 2003; Lader et al., 2004). Thus, the lack of effectiveness of PAR in extinction training may reflect an insufficient dose used in WKY rats.

In summary, this study examined the effects of three classes of psychotropic agents commonly used in treating anxiety and depression-like symptoms in humans on extinction of a lever-press active-avoidance task in rats. Given the behavioral, neurochemical, and pharmacological features demonstrated in the WKY rat, NET, and DAT inhibitors were more effective in facilitating extinction of avoidance behaviors but SSRIs was the least effective. Thus, the WKY rat could be used as a powerful tool to examine novel treatment targeting anxiety symptoms in patient population that is resistant to conventional SSRI treatment. Similar to the enhanced prevalence of anxiety disorder in females (Pigott, 2003), we have reported that female SD rats are more sensitive to learn avoidance than male SD rats, while female and male WKY rats learn avoidance to similar degrees (Beck et al., 2011). It would be important to assess the effects of monoaminergic drugs on female rats in the future.

## Conflict of Interest Statement

The authors declare that the research was conducted in the absence of any commercial or financial relationships that could be construed as a potential conflict of interest.
